# A hybrid type I trial to increase Veterans’ access to insomnia care: study protocol for a randomized controlled trial

**DOI:** 10.1186/s13063-017-2437-y

**Published:** 2018-01-26

**Authors:** Adam D. Bramoweth, Anne Germain, Ada O. Youk, Keri L. Rodriguez, Matthew J. Chinman

**Affiliations:** 1 Center for Health Equity Research and Promotion, VA Pittsburgh Healthcare System, Research Office Building (151R-U), University Drive C, Pittsburgh, PA 15240 USA; 2 Mental Illness Research, Education and Clinical Center, VA Pittsburgh Healthcare System, Research Office Building (151R-U), University Drive C, Pittsburgh, PA 15240 USA; 3 Department of Psychiatry, University of Pittsburgh, 3811 O’Hara Street, Pittsburgh, PA 15213 USA; 4 Department of Biostatistics, Graduate School of Public Health, University of Pittsburgh, Pittsburgh, PA USA; 5 Division of General Internal Medicine, Department of Medicine, University of Pittsburgh, Pittsburgh, PA USA; 6 RAND Corporation, 4570 Fifth Avenue, Suite 600, Pittsburgh, 15213 PA USA

**Keywords:** Insomnia, Randomized controlled trial, Cognitive behavior therapy, Behavior therapy, Qualitative research, Veterans

## Abstract

**Background:**

Chronic insomnia is among the most reported complaints of Veterans and military personnel referred for mental health services. It is highly comorbid with medical and psychiatric disorders, and is associated with significantly increased healthcare utilization and costs. Evidence-based psychotherapy, namely Cognitive Behavioral Therapy for Insomnia (CBTI), is an effective treatment and recommended over prescription sleep medications. While CBTI is part of a nationwide rollout in the Veterans Health Administration to train hundreds of providers, access to treatment is still limited for many Veterans due to limited treatment availability, low patient and provider knowledge about treatment options, and Veteran barriers such as distance and travel, work schedules, and childcare. Uptake of a briefer, more primary-care-friendly treatment into routine clinical care in Veterans Affairs (VA) primary care settings, where insomnia is typically first recognized and diagnosed, may effectively and efficiently increase access to effective insomnia interventions and help decrease the risks and burdens related to chronic insomnia.

**Methods:**

This hybrid type I trial is composed of two aims. The first preliminarily tests the clinical non-inferiority of Brief Behavioral Treatment for Insomnia (BBTI) versus the current “gold standard” treatment, CBTI. The second is a qualitative needs assessment, guided by the Consolidated Framework for Implementation Research (CFIR), to identify potential factors that may affect successful implementation and integration of behavioral treatments for insomnia in the primary care setting. To identify potential implementation factors, individual interviews are conducted with the Veterans who participate in the clinical trial, as well as VA primary care providers and nursing staff.

**Discussion:**

It is increasingly important to better understand barriers to, and facilitators of, implementing insomnia interventions in order to ensure that Veterans have the best access to care. Furthermore, it is important to evaluate the potential for new avenues of treatment delivery, like BBTI in the primary care setting, which can benefit Veterans who may not have adequate access to specialty mental health providers trained in CBTI.

**Trial registration:**

ClinicalTrials.gov, ID: NCT02724800. Registered on 31 March 2016.

**Electronic supplementary material:**

The online version of this article (doi:10.1186/s13063-017-2437-y) contains supplementary material, which is available to authorized users.

## Background

Chronic insomnia—difficulty initiating and maintaining sleep that persists for more than 3 months—is a prevalent disorder among adults, approximately 25%, but can be particularly pervasive among military personnel and Veterans, with estimates nearing 75% in some samples [1–5]. Chronic insomnia is among the most reported complaints of Veterans [6] and is the most common initial complaint of military personnel referred for mental health services [1, 7]. Potential risk factors include deployment overseas, engaging in combat, 24 hours/7 days a week work schedules, adjusting to separation from military and reintegration to civilian life, as well as the numerous medical and mental health problems that commonly affect military personnel and Veterans [8–13]. It is also a risk factor for the development of depression [14] and metabolic and cardiovascular diseases [15]. Furthermore, insomnia is associated with significant healthcare utilization, and both individual and societal economic burden [16–19].

Despite the significant impact of insomnia, it remains undertreated [20]. When identified, it is most often treated in primary care with pharmacotherapy, rather than the first-line recommendation, Cognitive Behavioral Therapy for Insomnia (CBTI) [21, 22]. Pharmacotherapy is associated with risks of dependence, tolerance, and poorer quality sleep [23–25], whereas evidence-based psychotherapies for primary and comorbid insomnia results in better long-term outcomes, no drug dependence or polypharmacy risk, and potential cost savings [26, 27]. Based on our experience, numerous system-, provider-, and patient-level factors contribute to the gap between the high prevalence of insomnia and the relatively low use of CBTI, and potentially contribute to the high use of prescription medications: (1) shortage of CBTI-trained clinicians; (2) treatment restricted to mental health clinics; (3) insomnia being considered a symptom of another disorder; (4) lack of patient and provider knowledge regarding CBTI availability; (5) barriers to attend appointments such as distance to travel, work schedule, and childcare; and (6) burdensome duration and delivery method (CBTI can be six or more in-person, 45-min sessions).

The Department of Veterans Affairs (VA) nationwide CBTI rollout, which began in 2011, substantially increased the number of providers who can deliver evidence-based treatment with fidelity and helped to increase access to care. An evaluation of 696 Veterans who participated as part of the CBTI rollout found that 60% who completed treatment had insomnia severity reductions, per the Insomnia Severity Index (ISI), of ≥ 8 points (mean change 20.7 to 10.9), with a pre- to post-treatment Cohen’s *d* effect size of 2.3 [28, 29]. While the rollout has been successful to date and continues to train providers, CBTI is still only being delivered to a fraction of those who could benefit. Thus, in order to increase the viability of cognitive and/or behavioral insomnia treatments in the VA, it is critical to not only determine which treatments are most effective, but also to determine which implementation factors (e.g., barriers and facilitators) most impact the uptake of these treatments by patients and providers in routine clinical practice. An evidence-based behavioral insomnia treatment that combines brevity (four sessions or fewer), multiple delivery modalities (in-person and phone), and is delivered by non-physician, non-sleep-specialist clinicians may help to overcome barriers associated with the current standard of care treatment, CBTI. However, the four weekly sessions of Brief Behavioral Treatment for Insomnia (BBTI) [30, 31] (two in-person, two phone calls), focused on the behavioral aspects of CBTI, have also proven to be efficacious among Veterans [32], and are potentially easier to implement in primary care settings because this approach is shorter and requires less training to deliver competently. Thus, BBTI could be an ideal intervention for delivery in the context of co-located, collaborative, integrated primary care within the VA, which employs a variety of providers of differing training levels.

Uptake of BBTI into primary care could effectively and efficiently increase access to insomnia treatment and potentially decrease some of the risks and burdens associated with chronic insomnia. However, it is necessary to determine whether BBTI offers non-inferior treatment outcomes to CBTI. Additionally, given that the implementation factors associated with BBTI and CBTI are not well known, it is also important to determine whether BBTI experiences fewer patient-, provider-, and system-level barriers to implementation than CBTI. Therefore, the current proposal utilizes a hybrid type I research design that includes: (1) a pilot comparative effectiveness trial of BBTI versus CBTI and (2) a qualitative needs assessment of healthcare provider- and Veteran-level implementation factors guided by the Consolidated Framework for Implementation Research (CFIR), the predominant model of implementation factors [33].

## Methods

### Overview and study design

**Fig. 1:**
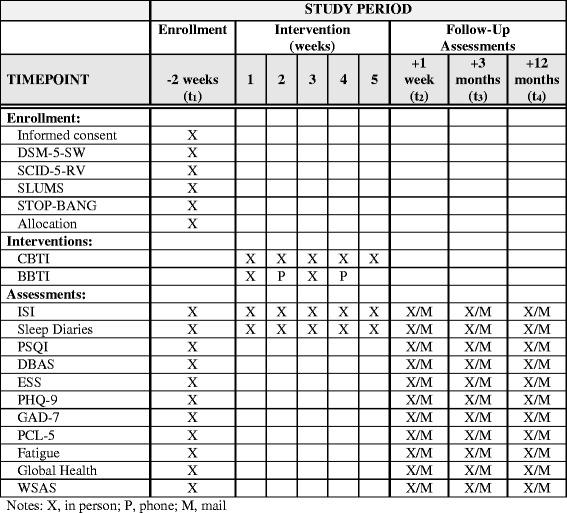
Schedule of enrollment, interventions, and assessments

This project is a hybrid type I, comparative effectiveness trial that compares BBTI to CBTI. The project is being conducted at one large, urban VA Medical Center (VAMC) over a 3-year period and is composed of two aims. The first aim is a randomized, non-inferiority trial to compare the effectiveness of BBTI versus CBTI. We hypothesize that (1) both BBTI and CBTI will significantly reduce insomnia symptoms, per the ISI, from pre- to post-treatment and (2) BBTI will be non-inferior to CBTI based on ISI change scores from pre- to post-treatment. Veterans with chronic insomnia are randomly assigned to either BBTI or CBTI with treatment being delivered by licensed psychologists and assessments at baseline, post-treatment, 3-month follow-up, and 12-month follow-up. See Fig. 1 for the Standard Protocol Items: Recommendations for Interventional Trials (SPIRIT) guidelines; the full SPIRIT Checklist is available as Additional file 1. CBTI is a well-established, evidence-based psychotherapy with broad dissemination throughout the VA and is considered the recommended first-line treatment for insomnia per the National Institutes of Health and the American College of Physicians [22, 34]. Treatment outcomes for CBTI typically include significant reduction of symptoms per the ISI and Cohen’s *d* effect sizes > 1 at post treatment [29]. The comparison treatment, BBTI, is also effective, with significant treatment outcomes similar to CBTI in active-duty military and Veterans’ samples [30, 32]; however, the two have not yet been compared directly.

The second aim is a qualitative needs assessment, guided by the CFIR, to identify potential factors that may impact successful implementation and integration of behavioral and cognitive behavioral treatments for insomnia in the primary care setting. Given the under-diagnosis of insomnia and subsequent inadequate resources to treat insomnia with behavioral and cognitive behavioral treatments, the qualitative needs assessment may identify key perceived barriers and facilitators to implementing these treatments in primary care, a setting that may be able to significantly increase access to treatment, especially when it is not available in other settings such as mental health and/or sleep medicine. Individual interviews are conducted with the Veterans who participate in the clinical trial, as well as primary care providers and nursing staff.

## Procedures, participants, measures, and analyses by study aim

### Aim 1: non-inferiority, comparative effectiveness trial of CBTI versus BBTI

#### Data source

Fifty-six Veterans with chronic insomnia will be randomized to either of the two treatment arms, BBTI (active comparison) or CBTI (reference treatment).

#### Recruitment and participants

The target population is a clinical sample of Veterans with chronic insomnia (see Table 1). Veterans are eligible if they meet the diagnostic criteria for an insomnia disorder according to the *Diagnostic and Statistical Manual of Mental Disorders-5* (DSM-5) and are 18 years of age or older. In an attempt to be as pragmatic and reflective of typical clinical samples, exclusionary criteria were limited to key factors that would likely prevent a Veteran from engaging in similar treatments for insomnia in a typical VA setting. Veterans are excluded if they have a disorder that would significantly increase their risk of experiencing side effects with standard treatment or would require a significant adaptation of treatment. Veterans are also excluded consistent with the standard of care for cognitive and/or behavioral treatment of insomnia at the study VAMC.

**Table 1:** Inclusion and exclusion criteria

Inclusion criteria
Military Veteran
Age ≥ 18 years
ISI ≥ 15 (moderately severe insomnia) and DSM-5 criteria for insomnia disorder
Exclusion criteria
Current/past bipolar disorder or seizure disorder, and current psychotic disorder in order to avoid potential exacerbation of manic/hypomanic, seizure, and psychotic symptoms, respectively, as adverse reactions to aspects of BBTI/CBTI (e.g., sleep restriction)
Current alcohol use disorder or substance use disorder as BBTI/CBTI cannot reverse the adverse effects of substances on sleep
Other current, severe or unstable, psychiatric and medical disorders that necessitate clinical management that can confound results (e.g., cancer [receiving chemotherapy], suicidality, recent hospitalization [medical/surgical] for which recovery overlaps with study onset and duration, open skull/brain injury, moderate to severe traumatic brain injury)
Previously diagnosed sleep apnea that is not adequately treated or probable sleep apnea (STOP-BANG ≥ 5)
Moderate to severe cognitive impairment (SLUMS ≤ 20) and/or diagnosis in the medical record indicative of moderate-to-severe cognitive impairment
If using a sleep medication, the medication and dose has changed in the past month and/or is expected to change during the treatment phase of the study
If using other psychotropic medication, medication and dose has changed in the past 2 months and/or are expected to change during the treatment phase of the study
The following are exclusionary as they represent (potentially) temporary states/situations that may significantly impair normal sleep:
Women who are pregnant and/or breast feeding
Unstable environment that is not in one’s control (e.g., homeless, temporary group home, care-taking duties at night)
Shift work: severe delayed sleep phase disorder (e.g., habitual bedtime after 4 a.m. or habitual rise time after 11 a.m.)
Restless leg syndrome with symptoms > 2 times per week and causing significant distress

*BBTI* Brief Behavioral Treatment for Insomnia, *CBTI* Cognitive Behavioral Therapy for Insomnia*, DSM Diagnostic and Statistical Manual of Mental Disorders*, *ISI* Insomnia Severity Index*,* *SLUMS* St. Louis University Mental Status Examination

#### Screening process

The screening interview, conducted by the study coordinator, includes gathering Veteran information regarding: demographics (age, sex, and Veteran status); clinical information, including a brief description of sleep complaint and the ISI (≥15, indicative of at least moderately severe insomnia); exclusionary DSM-5 diagnoses (e.g., psychotic disorders); known medical and sleep disorders; and current alcohol and drug use. This information is used to determine preliminary eligibility and those Veterans who are eligible are invited to a face-to-face visit to complete informed consent, a brief cognitive screen, a diagnostic assessment for sleep and psychiatric disorders, and collection of baseline measures. Ineligible Veterans, if interested, are referred to an appropriate clinic (e.g., behavioral health, sleep medicine, insomnia clinic). Once a participant is screened, they are assigned a unique study identification number that is used on all study documents in order to maintain confidentiality.

#### Baseline screening measures and assessments

The initial face-to-face visit includes answering Veterans’ questions and addressing concerns, and administering written informed consent and baseline assessments and measures. The study coordinator administers written informed consent and conducts the baseline assessment unless otherwise indicated.

In order to assess for insomnia disorder, inclusionary criteria, as well as other sleep disorders that may be exclusionary, the STRONG STAR Clinical Interview for DSM-5 Sleep-Wake Disorders (DSM-5-SW [35]) is used. The STRONG STAR Interview, developed by the South Texas Research Organizational Network Guiding Studies on Trauma and Resilience (STRONG STAR), was developed to assess for all DSM-5 Sleep-Wake disorders, including: insomnia, hypersomnia, circadian rhythm disorders, obstructive sleep apnea, restless leg syndrome, periodic limb movements, parasomnias, nightmares, rapid eye movement (REM) sleep behaviors, and narcolepsy. To further screen for the presence of untreated obstructive sleep apnea, participants are administered the STOP-BANG Questionnaire [36], which is an eight-item self-report screening tool for obstructive sleep apnea, scored yes/no. Scores range 0–8 with scores ≥ 5 indicative of high risk of moderate-to-severe obstructive sleep apnea. Questions ask about snoring, daytime sleepiness, blood pressure, Body Mass Index, age, neck circumference, and gender. Veterans scoring ≥ 5 are excluded from the study until further assessed by the study VAMC Sleep Medicine Clinic for sleep apnea or other breathing-related sleep disorders. Veterans with suspected non-insomnia sleep disorders are also referred to the Sleep Medicine clinic for further assessment. In the event that the Sleep Medicine Clinic evaluation rules out non-insomnia sleep disorders, the participant is eligible for the study.

To screen for potential exclusionary cognitive impairment, the St. Louis University Mental Status Examination (SLUMS [37]) is administered. The SLUMS is a brief cognitive impairment screening tool, validated in Veterans. Domains include: orientation; short-term memory recall; executive function; attention, concentration, and working memory; semantic fluency; and visuospatial ability. A score ≤ 20, indicative of serious cognitive impairment or dementia, results in exclusion from the study.

In order to establish the presence of psychiatric disorders as baseline participant characteristics and also as potential variables to include in analyses, participants are administered the Structured Clinical Interview for DSM-5 Research Version (SCID-5-RV [38]). The SCID-5-RV is a semi-structured interview guide for diagnosing DSM-5 disorders. It includes the major diagnostic categories and the diagnostic elements needed for inclusion and exclusion criteria. Assessing for a broad range of psychiatric disorders helps to better characterize the sample. The study principal investigator (PI), a licensed psychologist, conducts the SCID-5-RV assessment.

#### Outcome measures

The primary sleep outcome is the Insomnia Severity Index (ISI [39]). The ISI is a seven-item self-report measure of subjective insomnia severity, satisfaction with sleep, and daytime impairment. Items are rated 0–4; a cutoff of ≥ 15 points reflects clinically significant insomnia and is the minimum score to be eligible for participation. At post treatment, a reduction of ≥ 8 points indicates a moderate response to treatment; a score ≤ 7 points indicates no clinical insomnia and combined with a reduction of ≥ 8 points indicates treatment remission. The ISI is also administered at all in-person treatment sessions for both treatment arms. Since sleep is a multidimensional behavior, several additional self-report measures are used as secondary outcomes to assess changes pre to post treatment. We use Sleep Diaries [40] to measure sleep behaviors such as sleep onset latency (SOL), wake after sleep onset (WASO), sleep efficiency (SE), nighttime awakenings (NWAK), time in bed (TIB), total sleep time (TST), as well as to monitor bed times and wake times throughout treatment. The Dysfunctional Beliefs and Attitudes about Sleep (DBAS [41]) measures changes in sleep-disruptive cognitions and beliefs across treatment. The Pittsburgh Sleep Quality Index (PSQI [42]) is a measure of general sleep quality and the Epworth Sleepiness Scale (ESS [43]) measures daytime sleepiness.

Additional secondary outcome measures focus on self-reported psychiatric, psychosocial, and quality of life parameters. Psychiatric symptoms are assessed with the Patient Health Questionnaire 9 (PHQ-9 [44]) for depressive symptoms, the Generalized Anxiety Disorder 7 (GAD-7 [45]) for anxiety symptoms, and the PTSD Checklist for DSM-5 (PCL-5 [46]) for symptoms of posttraumatic stress disorder. Fatigue is measured by the Patient-Reported Outcome Measurement Information System (PROMIS) fatigue scale [47]. The PROMIS Global Health Scale [48] measures overall quality of life and the Work and Social Adjustment Scale (WSAS) [49] measures psychosocial functioning. Each of these outcome measures is administered at baseline, post-treatment, 3-month follow-up, and 12-month follow-up (see Fig. 1). To the extent possible, the research team attempts to collect outcome measures at the appropriate time points for all participants, even those who drop out of treatment.

#### Randomization

Following the diagnostic assessment and confirmation of eligibility, 56 Veterans are randomized in a 1:1 manner to the two treatment groups, BBTI (*N* = 28) or CBTI (*N* = 28). Randomization is at the patient level, stratified by age (18–64, 65 + years) and if taking a prescription sedative hypnotic medication (yes/no), with a 1:1 allocation using random block sizes of 2, 4, or 6. Assignments are generated by the study statistician and placed in opaque, sealed envelopes. The study coordinator is responsible for informing the Veterans of their treatment group assignment. The study PI is blinded to participant allocation but may need to be unblinded for clinical reasons (e.g., participant dropout and seeking clinical referral for treatment).

#### Clinical interventions

CBTI [50] is a structured treatment that addresses sleep-related behaviors and cognitions; it is effective in reducing insomnia severity and improving sleep quality, and is the standard non-pharmacological treatment for insomnia in VA. As part of this study, CBTI consists of five in-person sessions (weekly or biweekly) and is delivered by two licensed psychologists, trained in CBTI. The behavioral aspects of the treatment include sleep restriction (to increase the sleep drive and consolidate sleep through decreasing SOL and WASO) and stimulus control (to strengthen the bed as a cue for sleep and remove non-sleep stimuli from the bedroom environment) and may also include relaxation exercises (to reduce physiological arousal). The cognitive component focuses on restructuring or changing maladaptive sleep-related cognitions, such as thoughts that increase cognitive arousal and beliefs that interfere with adherence to the behavioral aspects of insomnia treatment.

BBTI [51] is a structured treatment that focuses on sleep-related behaviors and is effective for the treatment of insomnia. The behavioral aspects of the treatment, similar to those in CBTI, have been shown empirically to improve sleep quality through sleep restriction and stimulus control. Unlike CBTI, there are no cognitive components to treatment. BBTI is delivered over four consecutive weeks, consisting of two in-person sessions (weeks 1 and 3) and two phone sessions (weeks 2 and 4). The therapist manual and participant workbook were adapted for Veterans from the initial treatment protocols [51]. Treatment is delivered by two licensed psychologists, trained in BBTI.

Study clinicians are rated for treatment fidelity and competency using the CBTI rating scale from the VA CBTI Therapist Manual. BBTI clinicians are rated on an adapted rating scale. Treatment sessions are rated based on audio-recordings on a monthly basis with a random sample of each clinician rated. If study clinician’s ratings are below the cut-off (<28, CBTI; <22, BBTI) the study PI provides additional training to the clinician. Discontinuation from treatment is voluntary per the participant or if deemed clinically indicated by the study clinicians.

#### Follow-up assessments

The self-report measures assessed at baseline are repeated following the end of their intervention (post treatment), at 3-months post-treatment, and again at 12-months post-treatment.

#### Hypothesis

We hypothesize that both BBTI and CBTI significantly reduce insomnia symptoms, per the ISI, from pre- to post-treatment. We also hypothesize that BBTI is a non-inferior (i.e., similar) treatment to CBTI based on ISI change scores from pre- to post-treatment.

#### Data analysis

To test the effectiveness of BBTI and CBTI, using an “intent-to-treat” approach, we will fit a linear mixed model that tests for the main effects of treatment group (BBTI versus CBTI) and time (baseline, post-treatment, 3-month, and 12-month follow-ups) as well as the interaction of treatment and time. A secondary analysis of treatment effectiveness includes calculating the rates of treatment response and remission, and calculating the number needed to treat (NNT) for BBTI and CBTI [30]. To determine treatment non-inferiority, the 95% confidence interval (CI) of the mean ∆ISI for the BBTI group is compared to a non-inferiority margin (NIM) [52, 53]. If the 95% CI is entirely to the right of the NIM, we will have shown non-inferiority [54]. The non-inferiority margin (NIM) represents the maximum acceptable difference between ∆ISI_BBTI_ and ∆ISI_CBTI_.

#### Power analysis

To achieve adequate power (power = 0.80, α = 0.05), a sample size of *n* = 42 (*n* = 21 per group) at post-treatment was determined using methods appropriate for non-inferiority trials [55]. Estimation of sample size was informed by the Reliable Change Index (RCI [56]), which was calculated using data from the VA CBTI rollout, a nationwide effort to train providers in the VA to deliver CBTI [29]. The RCI is a metric to help determine if the magnitude of change (e.g., pre- to post-treatment) is statistically reliable. For this comparative effectiveness trial, RCI represents the change on the ISI from pre- to post-treatment (CBTI) that would be expected by chance. An RCI > 1.96 indicates that the post-test score likely reflects real change (versus the pre-test score) and the change is not due to chance [56]. The RCI used for power analysis, based on the pre- to post-CBTI ∆ISI, was RCI = 3.43. In order to reach *n* = 42, our goal is to randomize 56 Veterans (*n* = 28/group) given that we are estimating 25% withdrawal/dropout after randomization (based on the VA CBTI rollout [28, 29] and BBTI clinical trials [30, 32]).

### Aim 2: qualitative needs assessment to identify perceived barriers to, and facilitators of, implementation

#### Data source

Primary care providers, primary care nurses, and Veterans (from aim 1).

#### Recruitment and participants

The target sample is 8–12 primary care providers (physicians, physician assistants, and nurse practitioners) and 8–12 primary care nurses (registered nurse care managers and licensed practical nurses) from the study VAMC who treat patients with chronic insomnia. We use purposive sampling so that we can gather a range of perspectives from healthcare providers involved in the care process and Veterans involved in the clinical trial. To the best of our ability, we use the typical case strategy in order to highlight the general experience of primary care providers and nurses who treat patients with insomnia [57]. We also plan to use the snowball strategy so that those who we recruit and interview can help us identify additional similar providers and nurses in primary care who would also be informative [57]. These types of providers are targeted as they represent the front-line clinicians that Veterans typically engage with and report various health problems and symptoms to, including difficulty sleeping. It is valuable to know these providers’ thoughts, opinions, and perspectives in order to identify and better understand perceived barriers to, and facilitators of, implementation and to help improve access to care. Recruitment methods of primary care providers and nurses include invitation to participate by the PI based on established relationships through his work as a primary care-mental health integration psychologist. Also, primary care leadership is assisting with recruitment through the broad invitation to providers and nurses to participate in the research study by email. Also targeted for recruitment are 8–12 Veterans from each treatment group in aim 1 who completed at least one treatment session. Similar to the providers, the feedback from Veterans engaged in treatment will provide useful information to help improve access to care. However, the Veteran interview will differ from the provider interview with a focus on their experiences in treatment, opinions and perspectives on the treatment not received, and preferences for care. The goal of recruiting 8–12 per group is to achieve thematic saturation, when data collection and analysis reveals no new themes [58]. If thematic saturation is not achieved with 8–12 participants in each group, then we will continue with interviews until saturation is reached [58]. The study coordinator or other research staff administers written informed consent; the study coordinator, research staff, or PI conducts the qualitative interviews.

#### Measures and data collection

As mentioned above, the goal of this qualitative needs assessment is to identify perceived barriers to, and facilitators of, implementing behavioral treatment of insomnia into the primary care setting. Guiding the discovery and evaluation of implementation barriers is the CFIR [33]. The CFIR was developed with the specific goal of improving Veteran healthcare by implementing research findings into routine clinical practice. It is advantageous for initial implementation efforts as it combines numerous implementation theories and can be applied across a broad range of domains. CFIR is organized into five domains, each with numerous factors that may influence successful implementation; the five domains are: (1) intervention characteristics, including the strength and quality of treatments; (2) outer settings such as external policies and incentives to integrate treatment; (3) inner settings such as the culture of a clinic; (4) individual characteristics such as self-efficacy to engage in, and benefit from, treatment; and (5) the implementation process such as engagement of clinic providers in using a treatment. The CFIR domains and factors act as a guiding framework for implementation research.

The basic structure of the interview guide for the primary care providers and nurses is to introduce them to the basics of BBTI and CBTI and then ask a series of open-ended questions and discussion items related to CFIR factors, assessing any perceived barriers to, or facilitators of, implementing brief treatments for insomnia in the primary care setting. Veteran interviews focus on their experience in treatment, how it can further be improved, as well as a discussion about the treatment they received versus the treatment they did not.

#### Hypothesis

There are no specific a priori hypotheses. However, the goal of this qualitative aim is to identify potential provider- and Veteran-level factors, using a CFIR-guided qualitative needs assessment, which may impact successful implementation and integration of behavioral and cognitive behavioral treatments for insomnia in the primary care setting and help to improve access to care.

#### Data analysis

Using a constant comparative approach, data collection and analysis will be concurrent. Coding will use the Editing Style [59], which involves an open iterative coding approach that will allow for identification of basic concepts related to the CFIR factors as well as any additional factors that arise. As each interview is conducted and transcribed, transcripts are reviewed by two coders to identify emergent themes and develop preliminary codebooks based on CFIR coding methods; a codebook will be developed for the providers and nurses and a separate codebook for Veterans [60]. The process involves two coders reading each transcript a number of times to familiarize themselves with the content and categorize the data. The coders will compare newly gathered and previously collected data to identify emergent concepts, categories, themes and relationships in the data and develop an initial set of codes to be applied to subsequent transcripts. As additional transcripts are examined, codes are operationally defined, refined, and agreed upon by the coders. Similar or related codes are collapsed and large codes will, when needed, be separated into more refined and conceptually precise codes. The focus is on identifying specific perceived barriers to, and facilitators of, successful implementation using the CFIR domains and factors. The provider and nurse codebook, and the Veteran codebook are finalized after review of the first four to six transcripts per cohort (providers, nurses, and Veterans); however, we will remain receptive to potential new codes emergent in remaining transcripts [61]. Once the codebooks are compiled and consensus is reached for each cohort, the finalized codebooks are independently applied to all transcripts by the two coders. Throughout codebook development and the coding process, the two coders meet to compare coding and resolve any discrepancies through negotiated consensus [62]. A study team member and expert qualitative methodologist serves as an adjudicator to ensure codebook development consistency and help resolve coding conflicts. Final analysis will allow us to identify common themes within the groups (i.e., provider/nurse and Veteran).

## Discussion

As CBTI continues to be disseminated to providers across the VA, it is increasingly important to better understand barriers to, and facilitators of, successful implementation of insomnia treatment in order to ensure that Veterans have the best access to care. Furthermore, it is important to evaluate the potential for new avenues of treatment delivery, like BBTI, that can benefit Veterans who may not have adequate access to specialty mental health providers trained in CBTI. As described above, this hybrid type I project preliminarily tests the clinical non-inferiority of a briefer, primary care-friendlier treatment, BBTI, versus the current “gold standard” treatment for insomnia, CBTI. Furthermore, utilizing CFIR-guided qualitative interviews with primary care providers and nurses, this project also seeks to identify key perceived barriers to, and facilitators of, implementing BBTI, or similar treatments, into the primary care setting in order to improve access to care. For Veterans, the interviews will help to understand their experiences in care and their care preferences. These are two of the strengths of this hybrid type I project.

Additionally, if the clinical trial is successful, and BBTI is shown to be non-inferior to CBTI, there may be a pathway toward broader training of BBTI as a complementary treatment to CBTI, with training focused on primary care staff like nurses and social workers, especially in settings where psychologists are not easily accessible or available (e.g., community-based outpatient clinics). Alternatively, even if BBTI is not found to be non-inferior to CBTI, there are still several directions to pursue. For example, if BBTI is not non-inferior to CBTI but still results in a significant treatment response for some Veterans, BBTI may still be appropriate for widespread dissemination and play an important role in stepped-care treatment for insomnia throughout VA. Also, by conducting a hybrid trial, the process of implementing high-quality, evidence-based practices, like BBTI, may be accelerated by the valuable input gained through qualitative interviews with both providers and Veterans. The qualitative interviews afford the participants the opportunity to provide in-depth information about their perspectives and insights about improving access to care, including advantages, disadvantages, and ways to succeed and the potential pitfalls to avoid from the frontline clinicians, and the open-ended interview structure allows for participant-directed responses. The perspectives of providers and Veterans on how to successfully implement BBTI into primary care settings can help guide the development of methods around identified barriers. Lastly, our multidisciplinary team has the broad spectrum of health services expertise needed to conduct this research, including health psychology, behavioral sleep medicine, implementation science, and qualitative and quantitative methods. The team members can make a substantive contribution to what is known about the implementation of behavioral treatments for insomnia in the primary care setting and improving access to care for Veterans with chronic insomnia.

While this study has potential to improve access to care for Veterans with chronic insomnia, it is not without limitations. The study site is a single, urban VAMC that is currently adequately staffed to manage its Veterans with insomnia. This may limit the generalizability of results for both study aims, not only among other VAMCs but also for the general community. Conducting a multisite hybrid trial may have helped to solve these limitations and may be an appropriate design for future studies to confirm, expand, or explore alternatives based on the current study’s findings. In regards to limitations of qualitative research, the goal is not generalizability in a statistical sense, but it is important to assess the ability of the proposed research to generate findings with utility beyond merely describing the specific study settings and sample. Qualitative research has been shown to produce fine-grained and rich descriptive analysis not achievable with purely quantitative approaches, and generate hypotheses and theoretical insights that can be usefully extrapolated, tested, and implemented beyond the specific study settings and sample. Given the current lack of empirical data on BBTI versus CBTI as well as implementing brief behavioral treatments into the primary care setting, we argue that a qualitative approach to identifying providers’ perceived barriers to, and facilitators and Veterans’ experiences of, treatment and preferences for care, is well justified and will make a significant contribution to the existing knowledge base. We have chosen to include a primary care providers (i.e., physicians, physician assistants, and nurse practitioners), nurses (registered nurse care managers and licensed practical nurses), and Veterans in hopes of increasing variation in the perspectives of valuable stakeholders and maximizing our ability to identify a more exhaustive list of barriers to, and facilitators of, implementation.

CBTI currently has significant support as the recommended first-line treatment for chronic insomnia for adults [34]. Still, there is much work to be done to provide effective and accessible insomnia care to Veterans, as well as the general community, and the results of this hybrid study may help reach those goals.

This project is registered with ClinicalTrials.gov, ID: NCT02724800 (URL: https://clinicaltrials.gov/ct2/show/NCT02724800).

## Trial status

VAPHS Institutional Review Board (IRB) approval for this study was granted in December 2015. Funding from VA HSR&D began in April 2016. Recruitment was initiated in May 2016 with the first participant randomized in June 2016. The final participant randomization is expected in January 2019.

## Additional file

Additional file 1: SPIRIT guidelines Checklist. (DOC 120 kb)

## Abbreviations

BBTI: Brief Behavioral Treatment for Insomnia
CBTI: Cognitive Behavioral Therapy for Insomnia
CFIR: Consolidated Framework for Implementation Research
CI: Confidence interval
DBAS: Dysfunctional Beliefs and Attitudes about Sleep
DSM-5: *Diagnostic and Statistical Manual of Mental Disorders, 5th Edition*
DSM-5-SW: STRONG STAR Clinical Interview for DSM-5 Sleep-Wake Disorders
ESS: Epworth Sleepiness Scale
GAD-7: Generalized Anxiety Disorder Scale
IRB: Institutional Review Board
ISI: Insomnia Severity Index
NIM: Non-inferiority margin
NNT: Number needed to treat
PCL-5: PTSD Check List for DSM-5
PI: Principal investigator
PROMIS: Patient-Reported Outcomes Measurement Information System
PSQI: Pittsburgh Sleep Quality Index
PTSD: Posttraumatic Stress Disorder
RCI: Reliable Change Index
SCID-5-RV: Structured Clinical Interview for DSM-5, Research Version
SE: Sleep efficiency
SLUMS: St. Louis University Mental Status Examination
SOL: Sleep onset latency
SPIRIT: Standard Protocol Items: Recommendations for Interventional Trials
TIB: Time in bed
TST: Total sleep time
VA: Veterans Affairs
WASO: Wake after sleep onset
WSAS: Work and Social Adjustment Scale
